# Publisher Correction: Cause analysis of PM_2.5_ pollution during the COVID-19 lockdown in Nanning, China

**DOI:** 10.1038/s41598-021-94322-1

**Published:** 2021-07-27

**Authors:** Zhaoyu Mo, Jiongli Huang, Zhiming Chen, Bin Zhou, Kaixian Zhu, Huilin Liu, Yijun Mu, Dabiao Zhang, Shanshan Wang

**Affiliations:** 1 Shanghai Key Laboratory of Atmospheric Particle Pollution and Prevention, Department of Environmental Science and Engineering, Fudan University, No. 220 Handan Road, Shanghai, 200433 China; 2 Atmospheric Environment Research Center, Scientific Research Academy of Guangxi Environmental Protection, Nanning, 530021 China; 3 Department of Occupational Health and Environmental Health, School of Public Health, Guangxi Medical University, Nanning, 530021 China

Correction to: *Scientific Reports* 10.1038/s41598-021-90617-5, published online 27 May 2021

The original version of this Article contained errors in Table 1 and the accompanying table legend.

The Table headings indicating the light absorption coefficients **σ**_**abs**_ were incomplete and omitted the term (Mm^−1^).

The correct and incorrect headings appear below.

Incorrect:

**Table:** 

**Stages**	**σ**_**abs,370**_	**σ**_**abs,470**_	**σ**_**abs,520**_	**σ**_**abs,590**_	**σ**_**abs,660**_	**σ**_**abs,880**_	**σ**_**abs,950**_	**AAE**

Correct:

**Table:** 

**Stages**	**σ**_**abs,370**_ (**Mm**^**−1**^)	**σ**_**abs,470**_ (**Mm**^**−1**^)	**σ**_**abs,520**_ (**Mm**^**−1**^)	**σ**_**abs,590**_ (**Mm**^**−1**^)	**σ**_**abs,660**_ (**Mm**^**−1**^)	**σ**_**abs,880**_ (**Mm**^**−1**^)	**σ**_**abs,950**_ (**Mm**^**−1**^)	**AAE**

In addition, the Table legend was incorrectly given.

“Biogeographic region follows the biogeographic provinces in Bowen and others (2016). Climate data from www.climate-data.org, accessed October 2018. Tidal pattern data from data.shom.fr, accessed October 2018.”

now reads:

“Aerosol light absorption properties.”

The original Article has been corrected.

